# Comparing the Impact of Online Ratings and Report Cards on Patient Choice of Cardiac Surgeon: Large Observational Study

**DOI:** 10.2196/28098

**Published:** 2021-10-28

**Authors:** Xuan Li, Shin-Yi Chou, Mary E Deily, Mengcen Qian

**Affiliations:** 1 Capital One Financial Corporation McLean, VA United States; 2 Department of Economics Lehigh University Bethlehem, PA United States; 3 School of Public Health, Fudan University, Key Laboratory of Health Technology Assessment, Ministry of Health Shanghai China

**Keywords:** online physician reviews, report cards, cardiac surgeons, patient choice

## Abstract

**Background:**

Patients may use two information sources about a health care provider’s quality: online physician reviews, which are written by patients to reflect their subjective experience, and report cards, which are based on objective health outcomes.

**Objective:**

The aim of this study was to examine the impact of online ratings on patient choice of cardiac surgeon compared to that of report cards.

**Methods:**

We obtained ratings from a leading physician review platform, Vitals; report card scores from Pennsylvania Cardiac Surgery Reports; and information about patients’ choices of surgeons from inpatient records on coronary artery bypass graft (CABG) surgeries done in Pennsylvania from 2008 to 2017. We scraped all reviews posted on Vitals for surgeons who performed CABG surgeries in Pennsylvania during our study period. We linked the average overall rating and the most recent report card score at the time of a patient’s surgery to the patient’s record based on the surgeon’s name, focusing on fee-for-service patients to avoid impacts of insurance networks on patient choices. We used random coefficient logit models with surgeon fixed effects to examine the impact of receiving a high online rating and a high report card score on patient choice of surgeon for CABG surgeries.

**Results:**

We found that a high online rating had positive and significant effects on patient utility, with limited variation in preferences across individuals, while the impact of a high report card score on patient choice was trivial and insignificant. About 70.13% of patients considered no information on Vitals better than a low rating; the corresponding figure was 26.66% for report card scores. The findings were robust to alternative choice set definitions and were not explained by surgeon attrition, referral effect, or admission status. Our results also show that the interaction effect of rating information and a time trend was positive and significant for online ratings, but small and insignificant for report cards.

**Conclusions:**

A patient’s choice of surgeon is affected by both types of rating information; however, over the past decade, online ratings have become more influential, while the effect of report cards has remained trivial. Our findings call for information provision strategies that incorporate the advantages of both online ratings and report cards.

## Introduction

Since the early 1990s, federal and state agencies in the United States have published hospital report cards that measure the risk-adjusted performance of physicians so as to encourage quality-informed decisions by patients. Generally, complex mathematical tools are applied to inpatient records and clinical data to correct for patient characteristics and severity of conditions to produce arguably reliable and objective quality information, a process that is costly in time and resources. However, studies have found that patient use of this information is below expectation: although reductions in volume for poor-performing providers is observed, the size of the effect is small, and highly rated providers are not always rewarded with increased market share [1-4]. Possible reasons include low awareness [5], high cognitive burden of understanding and interpreting quality metrics [6], and the absence of information more interesting to patients, such as health gains, waiting time, or bedside manner [7].

Fortunately, the spread over the last decade of physician review platforms provides a new information source that patients can use when choosing a physician. These platforms (ie, Vitals, ZocDocs, and RateMDs) allow patients to assign a general rating, indicate detailed ratings of specific aspects of their experience, and write comments, which are then disclosed to the public (see Panel A of Figure S1 in Multimedia Appendix 1). In contrast to report cards, online reviews are prompt, straightforward, and more subjective.

Recent studies of the reliability of physician online ratings, however, show mixed results [8-11]. Some researchers argue that online ratings may not reflect actual quality information due to “fake” reviews and a lower response rate among patients with less favorable outcomes [12,13]. Further, while some researchers found that patients treated by cardiac surgeons with lower online ratings had lower odds of surviving [14], others found no correlation between online ratings and clinical quality measures [15,16].

However, despite being controversial as quality measures, online physician reviews may still affect patient choice. First, such ratings are popular: surveys suggest that 50% to 75% of US consumers choosing a physician consult physician review platforms [17,18]. Second, online reviews serve as a form of word-of-mouth. Previous studies suggest that the impact of market-based learning on patient choice is greater than public report cards, and that narratives from nonprofessionals have a powerful impact [19,20]. Unfortunately, few studies have directly examined the impact of online ratings on patient demand. Early attempts used the volume of appointments scheduled via review platforms as the outcome measure [21-24]. However, previous studies are subject to two major concerns: first, online appointments do not necessarily result in an actual office visit, which introduces measurement errors; second, good online ratings and the decision of providing online scheduling may be correlated with unobserved surgeon characteristics, resulting in selection of physicians [25].

In this study, we investigated patient responses to different quality information sources using both online ratings and report card scores. We took advantage of the quality reporting based on risk-adjusted mortality rates achieved by individual cardiac surgeons in Pennsylvania, United States, which the state publishes every 1 to 2 years. We compared the impact of online ratings on patient choice with that of report card scores by linking both types of rating information to inpatient discharges in Pennsylvania over a 10-year period.

## Methods

### Data and Variables

#### Inpatient Records

We used inpatient discharge records collected by the Pennsylvania Health Care Cost Containment Council (PHC4) to identify coronary artery bypass graft (CABG) surgeries (ICD-9-CM [International Classification of Diseases, Ninth Revision, Clinical Modification] procedure codes 36.10-36.19) performed from January 2008 through December 2017. Each record included the quarter of admission, patient characteristics (eg, age, sex, and home zip code), the operating physician’s license number, and a hospital identifier. We used the operating physician’s license number and the Pennsylvania State Licensing System to identify the physician’s full name and practice location, which were then used to identify the online ratings of the physician. In this step, we matched information for 99.4% of surgeons. We focused on surgeons who received at least one set of report card scores during our study time frame and obtained a sample of 37,354 CABG surgeries performed by 184 surgeons.

#### Online Physician Ratings

Our online ratings were from the website Vitals. Vitals was launched in 2008 and is a leading physician review platform in the United States. We chose Vitals for two important reasons. First, it is free for both patients and providers to use and, thus, has become very popular, accumulating reviews for over 1 million doctors and 165,000 facilities. In the first half of 2018, for example, over 940,000 people in United States visited it each month. The usage of Vitals in Pennsylvania ranked in the top quartile among all the states (Figure S2 and Table S1 in Multimedia Appendix 1 give more information about Vitals, showing variations in Vitals usage across states and specialties, respectively). Second, Vitals provides the date that each review was posted, allowing us to identify the average ratings that a patient would see at the time of his or her surgery.

Each review on Vitals includes a required overall rating, optional ratings of eight specific aspects of the patient’s experience (ie, ease of making appointments, promptness, friendliness of the staff, accuracy of diagnosis, bedside manner, time spent with the patient, whether there was appropriate follow-up, and wait time), and optional written comments. All ratings range from one to five stars. For each provider, Vitals displays all received reviews and provides a summary with the average and the distribution of the overall rating earned by the physician across all of his or her practice hospitals (see Panel B of Figure S1 in Multimedia Appendix 1). We identified surgeons who performed CABG in Pennsylvania during our study period and who were reviewed on Vitals using surgeons’ full names, specialties, and locations. All reviews posted before January 1, 2018, were scraped. A total of 1096 reviews for 132 out of 184 surgeons (71.7%) were obtained (see Figure S3 in Multimedia Appendix 1 for variations in rating volumes across years).

We calculated the average overall ratings at the time of a patient’s surgery at the surgeon-by-quarter levels and matched this information to inpatient records based on the surgeon’s license number and the patient’s quarter of admission. In a given quarter, ratings were the same for the same surgeon who practiced at multiple hospitals. Following the literature [14], we used a categorical form of the rating information to address the potential underrepresentation of low ratings on Vitals (see Figure S4 in Multimedia Appendix 1) while also taking into account surgeons with no ratings. We gave a surgeon a “high online rating” each quarter when the surgeon’s average rating, based on reviews posted up to the most recent quarter, was at least four stars. We also created an indicator that equals 1 if the surgeon had not yet been reviewed on Vitals.

#### Report Card Scores

We obtained report card scores for providers from the seven issues of PHC4’s Cardiac Surgery Report published during our study period. Table S2 in Multimedia Appendix 1 lists the publication dates and data collection periods for each issue. Report cards provide quality scores for each surgeon who performed at least 30 CABG surgeries during the data collection period, indicating whether they had mortality rates that were greater than expected, as expected, or lower than expected based on risk-adjusted health outcomes of their patients (see Figure S5 in Multimedia Appendix 1). We used the reported summary measure for each surgeon across his or her practice hospitals. Following the literature [3], we assumed that patients used the most recent report card to inform their decisions and, therefore, that the effective period of each report card was from its publication date to the publication date of the next report card. Accordingly, we linked the most recent report card information to inpatient records, based on the surgeon’s name. We created an indicator, “high report card score,” which equals 1 if the surgeon had lower than expected mortality rates on the most recent PHC4 report card. We generated a dummy variable for surgeons with no score in the most recent report card.

#### Control Variables

We obtained hospital characteristics from the 2010 American Hospital Association’s Annual Survey of Hospitals. These were the number of beds, whether the hospital was a member of the Council of Teaching Hospitals, whether it had a cardiology intensive care unit (CICU), and the hospital’s zip code. We calculated the distance between a patient’s zip code and the zip code of the admitting hospital to capture the proximity of surgeons to patients.

#### The Final Inpatient Sample

We restricted our sample to patients with fee-for-service plans since their choices are expected to be less restricted by insurance networks. The final inpatient sample with full information consisted of 12,521 CABG discharges performed by 184 surgeons.

### Empirical Strategy

We estimated a random coefficient logit model, also known as the mixed logit model, to characterize a patient’s choice of surgeon [26]. The model has become the preferred method in hospital and physician choice analyses for two reasons [3,27]. First, the model estimates random coefficients on choice characteristics, thus eliminating the need to assume independence of irrelevant alternatives, which is likely to be violated in a model of surgeon choice. Second, the model allows for random taste variation among patients so that we can better incorporate consideration of patient heterogeneity. This feature is particularly important in our study because patients may differ in their knowledge of and willingness to use information from online ratings and report cards.

We assumed that each patient chooses among a set of possible surgeon-hospital pairs (ie, the surgeon’s rating is the same across the different hospitals where he or she works), and the observed portion of utility is additively separable in hospital and surgeon characteristics. The utility of patient *i* for choosing hospital *j* and surgeon *k* at time *t* can be specified as follows:




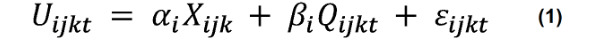




where *Q_ijkt_* is a vector of quality information variables and *X_ijk_* is a vector of observed hospital characteristics, including the patient’s distance from the hospital, and surgeon fixed effects, which capture the effect of time-invariant unobserved heterogeneity across physicians. *ε_ijkt_* is an idiosyncratic error term, which is assumed to be random with an independent and identically distributed extreme value distribution. Coefficients of the quality information and the distance variable were modeled as random variables with normal distributions to represent heterogeneity in patient tastes. The preference parameter, *β_i_*, is the parameter of interest, as it captures the marginal utilities of the different sources of quality information.

The probability that patient *i* chooses surgeon *k* conditional on *α_i_* and *β_i_* is as follows:




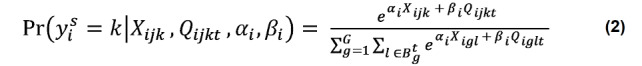




where *y_i_^S^* represents patient *i*’s choice of surgeon, *g* indexes the hospitals in patient *i*’s choice set, and *B_g_^t^* represents the set of surgeons available at hospital set *g* at time *t*. The unconditional choice probability can be then obtained by integrating the conditional choice probability from equation 2 over the probability density functions of *α* and *β* as follows:




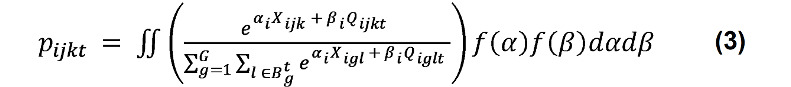




where *p_ijkt_* represents the probability of patient *i* choosing hospital *j* and surgeon *k* at time *t*, and *f* ( ) is the density function of a normal distribution. Through numeric integration, the log likelihood function of equation 3 can be maximized to yield estimates of the means and SDs of the preference parameters [28,29] .

To define the choice set, we drew a circle with its radius centered on a patient’s zip code and considered all surgeon-hospital pairs located within that circle as possible alternatives for the patient. In our main analysis, we followed the literature and used a radius of 50 miles, excluding discharges from patients who traveled beyond this cutoff [3], reducing the sample by 14.21% (1779/12,521). The estimation sample for the mixed logit models includes a total of 973,953 patient-alternative pairs.

Our variables of interest were the dummy variables for good quality information. We used two approaches to evaluate the impact of good ratings on patient choice. First, we calculated the average marginal effect (AME) of a surgeon receiving good ratings by doing the following: randomly selecting one surgeon from the choice set of each patient and bootstrapping 1000 times to calculate changes in the predicted probability of the surgeon being chosen when receiving a high rating versus not, then taking the average over all randomly selected surgeons.

Second, we calculated the impact of good ratings on patients’ willingness to travel (WTT). Because patients generally preferred a hospital closer to home, we used a patient’s WTT for a surgeon-hospital pair as an analog of the willingness to pay reported in preference studies involving prices. The WTT was calculated as the negative ratio of the estimated coefficient for high ratings to the estimated coefficient for distance.

### Robustness Checks

We performed four robustness checks. First, attrition of low-performing surgeons might lead to an overestimation of the effect of favorable quality information. We assume a surgeon has exited a patient’s choice set if he or she performed no CABG surgeries during the quarter that patient was admitted and no additional surgeries throughout the rest of our study period.

Second, we examined the effect of replacing the choice set based on a fixed 50-mile radius centered on the patient’s residence to a variable radius determined by the distance necessary to encompass 90% of patients who received CABG at a hospital (ie, the practice location of the surgeon). The variable radius captures the actual market of each practice hospital. Accordingly, we then redefined the choice set of a patient as surgeons whose practice hospitals had an actual market that covered the patient’s residence.

Third, we excluded patients with urgent and emergency conditions because these patients are less likely to have adequate time to gather information before making a choice. Finally, we considered the possibility that surgeons with good ratings also have better personal connections with primary care doctors or cardiologists and, thus, receive more referrals. As the inpatient records provide the license number of the doctors who made referrals, we restricted our sample to patients without this information in their records to address this concern.

## Results

### Descriptive Statistics

Table 1 reports descriptive statistics for key variables, first for all fee-for-service CABG discharges in Pennsylvania during our study time frame, and then for a sample restricted by travel distance. The average distance to the hospital dropped by more than half in the restricted sample due to the exclusion of patients who traveled more than 50 miles. All other observed characteristics were similar across the samples. In the restricted sample, at the time of a surgery, on average, 25.66% of surgeries were performed by surgeons with a high online rating, 68.43% by surgeons with no rating on Vitals, 6.04% by surgeons who received a high report card score, and 18.45% by surgeons with no report card scores.

**Table 1:** Descriptive statistics of the inpatient sample.

Characteristic		Inpatient sample without restrictions^a^ (N=12,521), mean (SD)	Inpatient sample restricted to discharges within 50 miles^b^ (N=10,742), mean (SD)
**Panel A: patient characteristics**			
	Age (years)	70.09 (9.39)	70.23 (9.32)
	Male (%)	69.79 (45.92)	69.66 (45.97)
	White (%)	86.64 (34.03)	85.88 (34.83)
	Nonemergency admission (%)	68.48 (46.46)	67.28 (46.92)
	Distance to hospital (miles)	32.94 (106.58)	14.17 (11.87)
**Panel B: surgeon characteristics (%)**			
	High online rating	25.57 (43.62)	25.66 (43.68)
	No online rating	68.60 (46.41)	68.43 (46.48)
	High report card score	5.85 (23.46)	6.04 (23.83)
	No report card score	18.34 (38.70)	18.45 (38.79)
**Panel C: hospital characteristics**			
	Teaching hospitals (%)	56.28 (49.61)	51.04 (49.99)
	Bed size, n	543.04 (359.77)	531.06 (355.82)
	Contains a cardiology intensive care unit (%)	93.09 (25.36)	92.09 (27.00)

^a^Inpatient sample consisting of fee-for-service patients who received a coronary artery bypass graft in Pennsylvania from January 2008 to December 2017.

^b^Subset of the above sample who traveled less than 50 miles.

### Main Results

Table 2 reports estimates of the random coefficient logit model. For each specification, we reported the estimated mean and SD of the effect of the quality information and travel distance. The estimated means represented the average responses of the patients in our sample, and the estimated SDs captured the degree of heterogeneity in such responses among patients. We found that the estimated mean of “high online rating” was positive and significant, suggesting that the average patient was more likely to choose a surgeon who had a higher online rating compared with lower-rated surgeons. In contrast, the estimated mean of “high report card score” was small and insignificant, suggesting no return in terms of patient preference for surgeons whose patients were less likely to die than expected.

**Table 2:** Random coefficient logit estimates: fee-for-service patients’ choices in Pennsylvania, United States^a^.

Variable	Mean (SE)	*P* value	SD (SE)	*P* value
High online rating	0.317 (0.058)	<.001	0.0643 (0.216)	.77
No online rating	0.536 (0.064)	<.001	1.015 (0.117)	<.001
High report card score	–0.057 (0.093)	.54	0.326 (0.381)	.39
No report card score	–0.516 (0.091)	<.001	0.828 (0.243)	<.001
Distance to hospital	–0.278 (0.005)	<.001	0.122 (0.004)	<.001

^a^The table presents random coefficient logit estimates from the estimation sample, which consists of within-50-mile alternatives for patients who received a fee-for-service coronary artery bypass graft in Pennsylvania, United States. The number of observations (n=973,953) is the number of patient-alternative pairs. Other control variables included hospital characteristics (ie, bed size, indicator for member of the Council of Teaching Hospitals, and indicator for having a cardiology intensive care unit) and surgeon fixed effects.

Our results suggest that patients responded differently to surgeons with no online ratings in Vitals and surgeons with no report card scores. The positive estimated mean of “no online rating” indicates that the average patient was more likely to choose a surgeon who had not been reviewed on Vitals compared to a surgeon who had been reviewed and received a low rating. The findings suggested that it was worse for a surgeon to appear on Vitals with a low rating than to not appear at all. In contrast, the estimated mean of “no report card score” was negative. Since the cardiac report cards assigned a rating to all surgeons performing at least 30 surgeries during the data collection period, the finding implied a desire by patients to avoid surgeons that performed fewer operations.

We found little heterogeneity among patients with respect to a surgeon having a high online rating or a high report card score: all patients viewed these positively. Patient heterogeneity was statistically significant, however, for missing online ratings or report card scores. The significant estimated SDs for “no report card score” and “no online rating” suggested that some patients may treat the absence of such information as a signal of lower quality, while others may not. Based on the cumulative standard normal distribution, we found that 70.13% (*ϕ* [0.536/1.015]) of patients considered no information better than low ratings on Vitals, while the other 29.87% thought the opposite. The corresponding figures were 26.66% and 73.34% (*ϕ* [0.516/0.828]) for “no report card score,” suggesting a slightly more unanimous opinion that a missing report card score was a negative signal.

We calculated AMEs and WTT for a high online rating to further understand the impact of this information. We found that holding other observables constant, a surgeon’s probability of being chosen would increase by 0.69 percentage points, on average, if the surgeon’s online rating changed from low to high. We defined the baseline probability of being chosen as the average ratio of the total number of selected surgeons over the total number of surgeons within patients’ choice sets. The above AME estimates of “high online rating” corresponded to a 22.47% increase in choice probability, compared with the baseline. In terms of WTT, in our sample an average patient was willing to travel 0.59 miles to reach a surgeon with high ratings on Vitals.

### Additional Analyses

Table 3 presents results of the robustness checks. The first column reports estimates from a sample restricted to surgeons who remained in the market for the entire 10-year period. The second column presents results using the variable radius approach to define the choice sets. The third column reports results from a sample excluding urgent and emergency admissions. The fourth column displays results for nonreferral admissions only. All of these results were qualitatively similar with those presented in Table 2, suggesting that our main results were robust to surgeon attrition, alternative choice set definitions, and emergency status of admissions, and that our findings were not driven by a referral effect.

Although the marginal effect of a high online rating is more influential than a high report card score on patient utilities on average, the relative importance of the two information sources to patient choices may have changed over time. The number of online reviews on Vitals for cardiac surgeons operating in Pennsylvania was relatively small until 2014; since then, they have become increasingly popular (Figure S3 in Multimedia Appendix 1). We explore this further by adding a set of interactions between each rating variable and a linear year trend to equation 1 to capture any temporal pattern in the main effects of the rating information.

**Table 3:** Robustness checks^a^.

Variable			Excluding exited surgeons^b^		*P* value		Variable radius^c^		*P* value		Nonemergency^d^		*P* value		Nonreferral^e^		*P* value
**High online rating**																	
	Mean (SE)	0.167 (0.067)		.01		0.142 (0.072)		.050		0.164 (0.070)		.02		0.172 (0.068)		.01	
	SD (SE)	0.826 (0.278)		.003		0.721 (0.398)		.07		0.004 (0.322)		.99		0.368 (0.399)		.36	
**No online rating**																	
	Mean (SE)	0.505 (0.067)		<.001		0.543 (0.075)		<.001		0.458 (0.079)		<.001		0.447 (0.074)		<.001	
	SD (SE)	1.089 (0.195)		<.001		1.247 (0.218)		<.001		1.154 (0.126)		<.001		1.076 (0.160)		<.001	
**High report card score**																	
	Mean (SE)	0.016 (0.096)		.87		–0.060 (0.124)		.63		–0.073 (0.085)		.39		0.036 (0.138)		.79	
	SD (SE)	0.324 (0.396)		.41		0.650 (0.305)		.03		0.190 (0.429)		.66		0.445 (0.482)		.36	
**No report card score**																	
	Mean (SE)	–0.273 (0.045)		<.001		–0.278 (0.047)		<.001		–0.282 (0.055)		<.001		–0.316 (0.055)		<.001	
	SD (SE)	0.109 (0.273)		.69		0.053 (0.268)		.84		0.169 (0.252)		.50		0.199 (0.344)		.56	
**Distance to hospital**																	
	Mean (SE)	–0.282 (0.005)		<.001		–0.327 (0.008)		<.001		–0.245 (0.005)		<.001		–0.293 (0.006)		<.001	
	SD (SE)	0.126 (0.005)		<.001		0.189 (0.009)		<.001		0.102 (0.005)		<.001		0.134 (0.005)		<.001	

^a^All random coefficient logit models also included hospital characteristics (ie, bed size, indicator for member of the Council of Teaching Hospitals, and indicator for having a cardiology intensive care unit) and surgeon fixed effects.

^b^Each column is a separate regression. This column reports estimates from a sample restricted to surgeons who did not exit the market. The number of observations (ie, the number of patient-alternative pairs) is 798,150.

^c^This column reports results using the variable radius approach to define the choice sets. The number of observations is 354,226.

^d^This column reports results from a sample excluding urgent and emergency admissions. The number of observations is 498,903.

^e^This column reports results for nonreferral admissions only. The number of observations is 682,852.

**Figure 1:**
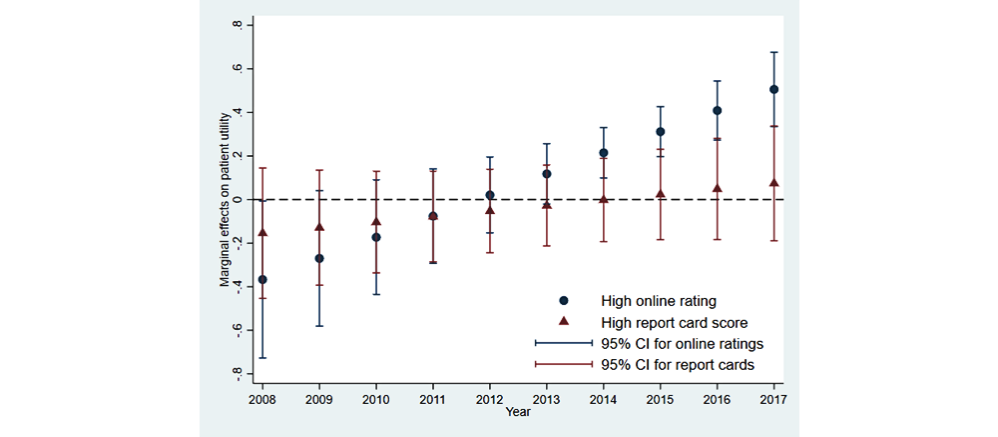
Changes in marginal effects of a high online rating and a high report card score on patient utility from 2008 to 2017. The marginal effects of a high online rating and a high report card score on patient utility are plotted with their 95% CIs by year. Calculations were based on estimates reported in Table 4.

Table 4 reports the results. The estimated means of the interaction of “high online rating” and a linear year trend were positive, while those of the interaction of “high report card score” and a linear year trend were around four times smaller and insignificant. Figure 1 depicts the marginal utilities of receiving a high online rating and a high report card score over time. The marginal effects of a high online rating were imprecisely estimated in the early years and then turned positive and significant after 2014. In contrast, the estimated marginal effects of a high report card score did not strengthen, remaining insignificant over our study period. Our results suggest that online ratings have become more influential for patient choice over time, while report card scores have not.

**Table 4:** Temporal variations in the main effects of quality information^a^.

Variable	Mean (SE)	*P* value	SD (SE)	*P* value
High online rating	–0.367 (0.184)	.046	0.106 (0.335)	.75
No online rating	–0.134 (0.213)	.53	0.840 (0.188)	<.001
High report card score	–0.154 (0.153)	.31	0.354 (0.357)	.32
No report card score	–0.907 (0.056)	<.001	0.052 (0.144)	.72
Distance to hospital	–0.277 (0.005)	<.001	0.122 (0.004)	<.001
High online rating × year	0.097 (0.027)	<.001	N/A^b^	N/A
No online rating × year	0.091 (0.033)	.005	N/A	N/A
High report card score × year	0.025 (0.024)	.29	N/A	N/A
No report card score × year	0.165 (0.012)	<.001	N/A	N/A

^a^The mean and SD columns present results from equation 1 with the interaction of quality variables and a linear time trend controlled. Estimation is based on discharges of fee-for-service patients who traveled no more than 50 miles. The number of observations (n=973,953) is the number of patient-alternative pairs. Other control variables included hospital characteristics (ie, bed size, indicator for member of the Council of Teaching Hospitals, and indicator for having a cardiology intensive care unit) and surgeon fixed effects.

^b^N/A: not applicable; these coefficients were not modeled as random variables.

## Discussion

This paper investigated the impact of online physician ratings on patients’ choice of CABG surgeon from 2008 through 2017 in Pennsylvania and compared it to the influence of report card scores. We estimated random coefficient logit models to allow for patient heterogeneity in preferences for surgeons with different ratings using a sample of fee-for-service patients to avoid impacts of insurance networks on patient choices. Our results show that positive assessments on the online physician rating platform had a significantly positive impact on a patient’s choice of surgeon, and that the impact increased over our study period. In contrast, the effect of a good report card score was trivial and did not change much over these years.

Two reasons may explain the increasing influence of online ratings. First, online ratings are much easier to use. If a patient searches for a surgeon, most of the returned links are to physician review websites. In contrast, to access report card scores, patients must know the scores exist, go to the correct website of the state agency, download the report, understand the meaning of the scores, and read through the report to find the physician that they are interested in. Second, online ratings can provide information on things like communication skills, friendliness of the staff, or ease of making an appointment, attributes that patients care about, but which are not available from report cards.

However, one concern about the increasing influence of online ratings is that the information provided on these review websites may steer patients away from surgeons who provide the best quality in terms of health outcomes. To shed light on this issue, we used text mining and machine learning techniques on the written comments on Vitals to identify attributes more likely to result in a higher overall rating. In our scraped reviews, about 13% of the ratings included written comments.

First, we calculated frequency of words and phrases. We found that phrases related to doctors’ attitudes toward patients, such as “bedside manner,” “doctor caring compassionate,” and “office staff,” were more frequently mentioned. In comments accompanying low ratings, patients often complained about scheduling issues and the time that the doctor spent with them. We then employed the classification and regression trees method to identify the strongest predictors of ratings among the frequently mentioned words [30]. Our results showed that the words “insurance,” “wait,” “appointment,” and “questions” were the strongest predictors of ratings. About 50% of the comments that contained “insurance” gave one star; around 79% of the comments that did not contain either “insurance” or “wait” gave five stars. This suggested that insurance was a purely negative factor: when insurance worked well, it did not contribute to high ratings, but when patients raised concerns about insurance-related issues with their surgeons, they were very likely to give them a low rating. Our findings implied that online ratings reflected more nonquality attributes of services than report cards.

Our study extends previous research regarding impacts of information on patient behaviors in several dimensions. First, this is the first study to compare the impacts of online ratings and public report cards on patient choice, providing insight into patient behavior when different types of quality information are available. Second, we presented new evidence on the impact of online ratings by examining inpatient volumes. Consistent with previous studies [21], we showed that online ratings exerted a positive effect on the popularity of physicians. However, previous literature generally relied on changes in volumes of virtual appointments to examine this issue. We, instead, linked the ratings directly to actual admissions and included in the analysis surgeons that had not been reviewed. Our approach helped avoid measurement error and selection of surgeons because online appointments do not necessarily end up with office visits and the absence of quality information may correlate with other surgeon characteristics. Third, we constructed a large sample that spans a 10-year period, allowing us to investigate changes over time in the relative importance of online ratings and report card scores for patient choice.

Our results yield important implications for both policy makers and online review platforms. Online ratings provide richer information about health services that patients seem to value and are becoming increasingly influential on patient choice. Nonetheless, such ratings are not driven by traditional quality indicators, and the report cards that do provide objective quality metrics have little impact. It is important to find ways to provide the health service market with information that incorporates the advantages of both online ratings and report cards. For policy makers, it is urgent to improve report card systems, for example, by making them easier to access and interpret, by working to increase media coverage of report card information, and perhaps by adding attribute measures that patients care about [1,27]. For online review platforms, it is important to work out ways to increase the importance of self-reported quality attributes in overall ratings.

Our study has several caveats. We discussed our results in terms of influences on patients’ choices, but we were not able to determine whether a patient had read online ratings or report cards before selecting a surgeon. Our results are, thus, better interpreted as an intent-to-treat effect. Moreover, although we found that a high online rating had a significant impact, we were not able to explore the mechanisms of the impact with our current data. For example, the subpopulation who are responsive to the online ratings information could be different, or online ratings may affect patient choices through younger members of a family. Understanding the underlying mechanisms is important and could be a focus of future research, because such knowledge would facilitate the design of systems that more effectively guide patients toward higher-quality providers.

## Appendix

Multimedia Appendix 1 Supplementary materials.

## Abbreviations

AME: average marginal effect
CABG: coronary artery bypass graft
CICU: cardiology intensive care unit
ICD-9-CM: International Classification of Diseases, Ninth Revision, Clinical Modification
PHC4: Pennsylvania Health Care Cost Containment Council
WTT: willingness to travel
