# Supplementary material for: Comparing the Impact of Online Ratings and Report Cards on Patient Choice of Cardiac Surgeon: Large Observational Study
Source: J Med Internet Res. 2021 Oct 28;23(10):e28098. doi: 10.2196/28098 (PMC8587194; doi:10.2196/28098)
Supplement: Multimedia Appendix 1 [file jmir_v23i10e28098_app1.docx]

**Supplementary Materials**

**Figure S1: An example of online physician review on vitals.com**


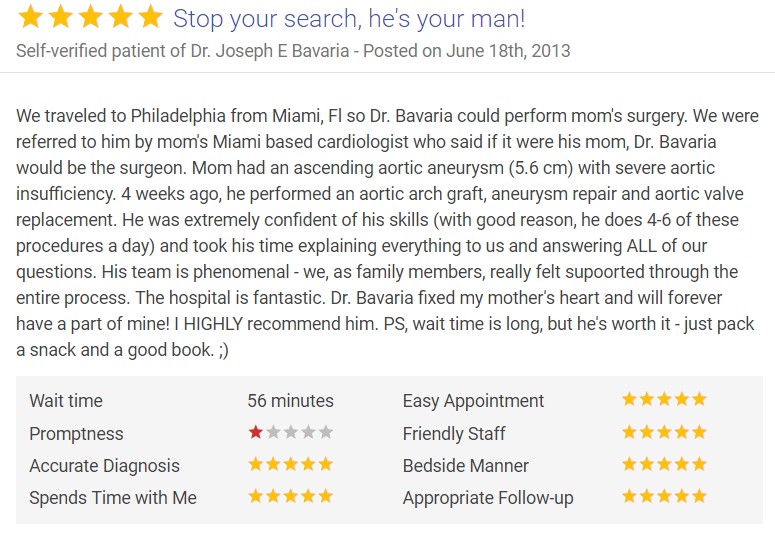


**Panel A: An example of individual review**


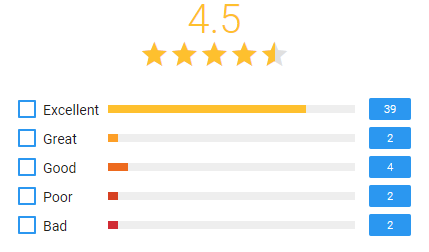


**Panel B: A summary of overall ratings for a doctor**

**Figure S2: Variations in the usage of Vitals based on a 5% sample of all listed doctors**


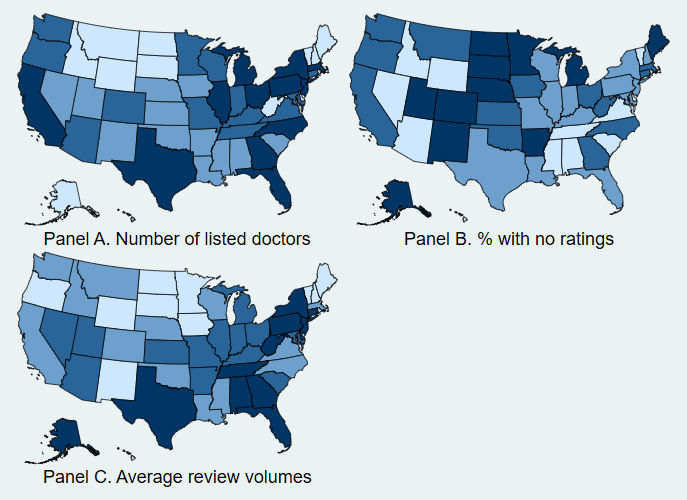


*N*otes: The information source is Vitals.com. From the physician directory page, we randomly selected 5% listed doctors and obtained their homepage links. We then scraped their names, practice state, specialty, and number of ratings received so far from their personal pages on Vitals. We used three measures to capture the usage of Vitals. These are the number of listed doctors, percent of doctors with no ratings, and the average review volumes of doctors who received at least one rating. For each of the measures, we divided states into quartiles and used a darker color to depict a higher value of the measure for a given state. Among all the states, we found that Pennsylvania ranked in the top quartile for the number of listed doctors and the average review volumes of doctors who received at least one rating; For percent of listed doctors with no ratings, Pennsylvania ranked below the median. Our results suggest that Vitals are popular in Pennsylvania.

**Figure S3: Online review volumes by years**


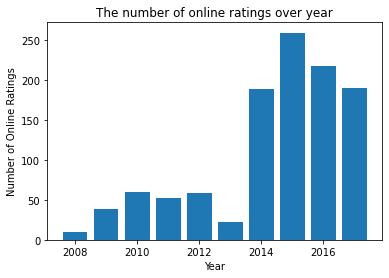


*Notes:* The data source is all reviews posted no later than December 2017 on Vitals.com for CABG surgeons who practice in Pennsylvania, US. We obtained a total of 1,096 reviews for 132 surgeons.

**Figure S4: The distribution of online rating scores**


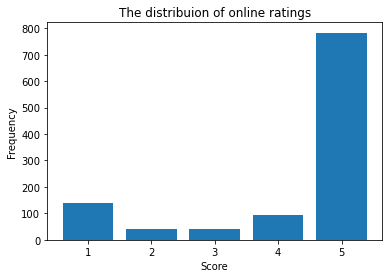


*Notes*: The data source is all reviews posted no later than December 2017 on Vitals.com for CABG surgeons who practice in Pennsylvania, US. We obtained a total of 1,096 reviews for 132 surgeons.

**Figure S5: A illustration of report cards rating**


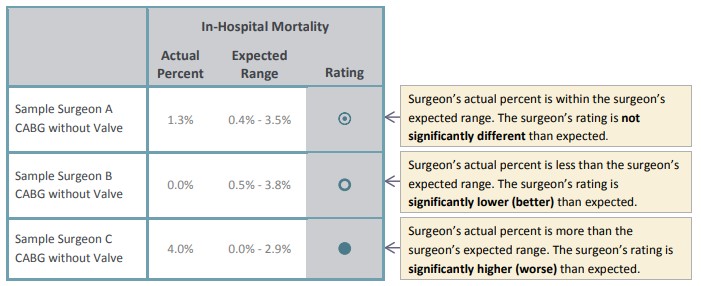


*Notes:* Information obtained from page 26 of PHC4 Cardiac Surgery Report, Jan 2014-Mar 2016.

**Table S1: Variations in Vitals usage across specialties, based on a 5% random sample**

| **Specialty** | **Listed physicians** | |  | **Rating information** | |
| --- | --- | --- | --- | --- | --- |
|  | **N** | **%** |  | **% with**  **no ratings** | **Average**  **Review volumes**  **if ever received**  **ratings** |
|  | (1) | (2) |  | (3) | (4) |
|  |  |  |  |  |  |
| Total | 75,257 | 100 |  | -- | -- |
| Internal Medicine | 13,151 | 17.62 |  | 36.19 | 10.86 |
| Cardiovascular diseases | 1,424 | 1.91 |  | 27.43 | 10.35 |
| Surgery | 8,813 | 11.80 |  | 53.75 | 18.79 |
| Orthopedic surgery | 4,714 | 6.31 |  | 67.27 | 24.50 |
| Vascular surgery | 435 | 0.60 |  | 30.77 | 11.89 |
| Cardiovascular-specific* | 17 | 0.02 |  | 5.88 | 6.88 |
| Psychiatry | 7,530 | 10.08 |  | 67.43 | 7.55 |
| Pediatrics | 4,998 | 6.69 |  | 45.74 | 7.91 |
| Obstetric and Gynecology | 2,671 | 3.58 |  | 20.00 | 18.16 |
| Family Medicine | 7,321 | 9.80 |  | 33.65 | 10.13 |
| Dentistry | 8,980 | 12.03 |  | 65.60 | 4.00 |
| Others | 15,203 | 19.56 |  | 58.74 | 10.30 |

*N*otes: The information source is Vitals.com. From the physician directory page, we randomly selected 5% listed doctors and obtained their homepage links. We then scraped their names, practice state, specialty, and number of ratings received so far from their personal pages on Vitals. The specialty of the doctors were classified according to the list of medical specialties and subspecialties provided by the following link <https://www.sgu.edu/blog/medical/ultimate-list-of-medical-specialties/>.

***** We note that our approach this approach fails to fully identify surgeons who provided cardiovascular-specific surgeries since most doctors do not provide subspecialty information at that level of detail on Vitals. Therefore, information listed in the table should be interpreted with caution. However, the issue is not a concern for our study because we are able to identify CABG surgeons from the inpatient records.

**Table S2: Pennsylvania cardiac surgery reports published 2007-2017**

| Publication year and quarter | Data collection period |
| --- | --- |
| (1) | (2) |
|  |  |
| 2007Q2 | 2005 |
| 2008Q3 | 2005-2006 |
| 2009Q3 | 2006-2007 |
| 2010Q3 | 2007-2008 |
| 2011Q2 | 2008-2009 |
| 2013Q4 | Jul 2011 - Dec 2012 |
| 2016Q1 | Jan 2014 - Mar 2016 |

### Notes: Information obtained from phc4.org/reports/cabg
